# Multi-detector CT angiographic imaging in the follow-up of patients after endovascular abdominal aortic aneurysm repair (EVAR)

**DOI:** 10.1007/s13244-012-0173-0

**Published:** 2012-06-12

**Authors:** Roberto Iezzi, M. Santoro, R. Dattesi, F. Pirro, M. Nestola, F. Spigonardo, A. R. Cotroneo, L. Bonomo

**Affiliations:** 1 Department of Bioimaging and Radiological Sciences, Institute of Radiology, “A. Gemelli” Hospital-Catholic University, L.go A Gemelli 8, IT-00168 Rome, Italy; 2 Department of Vascular Surgery, “G. D’Annunzio” University, Chieti, Italy; 3 Department of Bioimaging and Radiological Sciences, Institute of Radiology, “G. D’Annunzio” University, Chieti, Italy

**Keywords:** Endovascular abdominal aortic aneurysm repair (EVAR), CT imaging, Complications

## Abstract

**Background:**

Multidetector computed tomography (MDCT) angiography represents the standard of reference in the follow-up of patients after endovascular abdominal aortic aneurysm repair (EVAR), being effective in the detection of the full spectrum of possible complications on both axial and 3D images.

**Methods:**

The purpose of this article is to review the normal CT angiography findings of the different types of stent-grafts and to describe the radiological findings of early and late complications after EVAR on axial and reconstructed images. A selection of cases of post-EVAR MDCT angiography is presented to learn the techniques most commonly used for endovascular treatment, the correct CT scanning technique to acquire the data, the full gamut of possible procedure-related complications and how these complications usually appear on CT images.

**Conclusion:**

MDCT angiography is an effective and specific technique in both the pre- and postoperative settings of EVAR procedures. A better understanding of the procedure, the devices, the normal postoperative imaging features and the possible procedure-related complications ensures optimal planning and follow-up of patients undergoing an EVAR procedure.

## Introduction

Endovascular aortic aneurysm repair (EVAR) has developed into a feasible and successful alternative to open surgery for the treatment of abdominal aortic aneurysms. EVAR can be offered to many patients with a suitable anatomy of the aorta and iliac arteries, regardless of comorbid conditions [1–3]. Despite the known excellent early results of EVAR in terms of the reduction in perioperative mortality, rate of complications and length of hospitalization, many patients require re-intervention during the middle and long-term follow-up because of procedure-related complications. For this reason, surveillance of these patients is crucial to determine the long-term performance of these devices [4, 5]. Because of the rapid diffusion of EVAR and the increased number of patients who undergo multidetector CT (MDCT) follow-up, the radiologist should be familiar with the full spectrum of possible procedure-related complications in order to allow their early diagnosis and treatment. The purpose of this article is to present a spectrum of post-EVAR complications as seen with MDCT.

## Multidetector CT technique

**Fig. 1:**
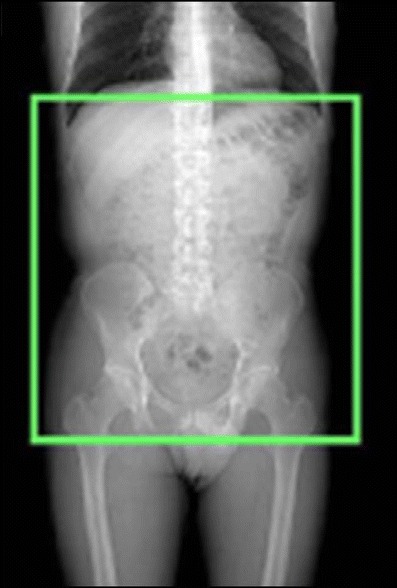
Scanning coverage of unenhanced and enhanced CT-acquisition

Regardless of the scanner available (4, 16 or 64 row), a thick-slice unenhanced acquisition can be performed to visualize the position of the stent-graft to reveal calcifications and to plan the following contrast-enhanced examination by selecting the acquisition volume and placing a region of interest in the aorta at the level of the celiac trunk (if bolus-tracking software is used). Contrast-enhanced images in the arterial phase are obtained during bolus intravenous injection of 90–130 ml of iodinated high-concentration non-ionic contrast medium, administrated with an automated injector at a flow rate of 3–4 ml/s through an antecubital vein. CT acquisition protocol parameters and scanning coverage ranges are illustrated Table 1 and Fig. 1. Scanning should start when the examined structures have reached an ideal level of opacification; therefore, the scan delay must be individualized to the patient by using bolus-tracking software to capture 100 HU on the abdominal aorta [6]. At the end of the arterial acquisition, delayed images focused on the graft must be acquired, performed at least 60 s and up to 120 s after contrast material injection. The CTA examination can be complemented by postprocessing reconstructions, including maximum- intensity projection (MIP), curvilinear reformation (CVR) and volume rendering (VR) [7].

**Table 1:** CT acquisition protocol parameters

Scanner	Rotation time (s)	Collimation	Table feed (mm/s)	Slice thickness (mm)	Slice interval (mm)	Duration (s)
4 slice	0.5	4 × 1 mm	25	1.25	1	25-30
16 slice	0.5	16 × 0.625 mm	27.5	0.625	0.625	25-30
64 slice	0.5	64 ×0.625 mm	80	0.625	0.625	<15

## Stent-grafts

**Fig. 2:**
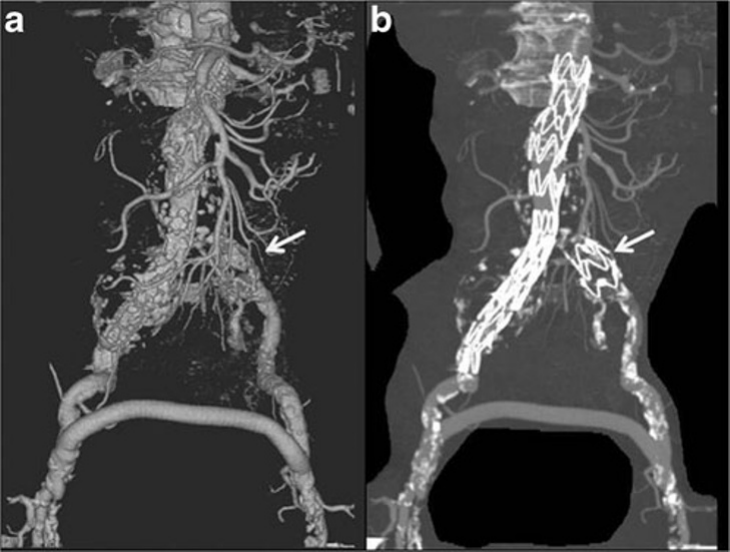
VRT (**a**) and MIP (**b**) images: aorto-uni-iliac device associated with a surgical femoro-femoral cross-over graft. The left iliac limb is occluded by an “occluder” (arrows)

**Fig. 3:**
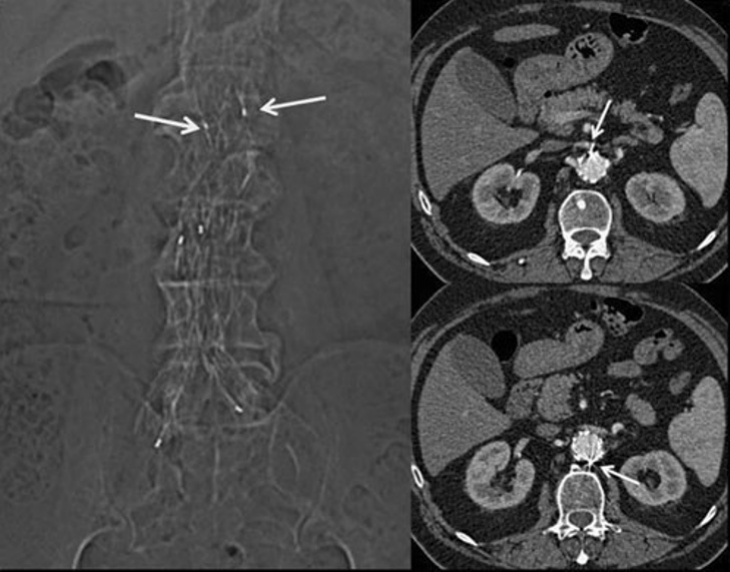
Scout view and axial images showing the two metallic radiological markers placed in the opposite site between the uncovered and covered portion in a suprarenal fixation device

**Fig. 4:**
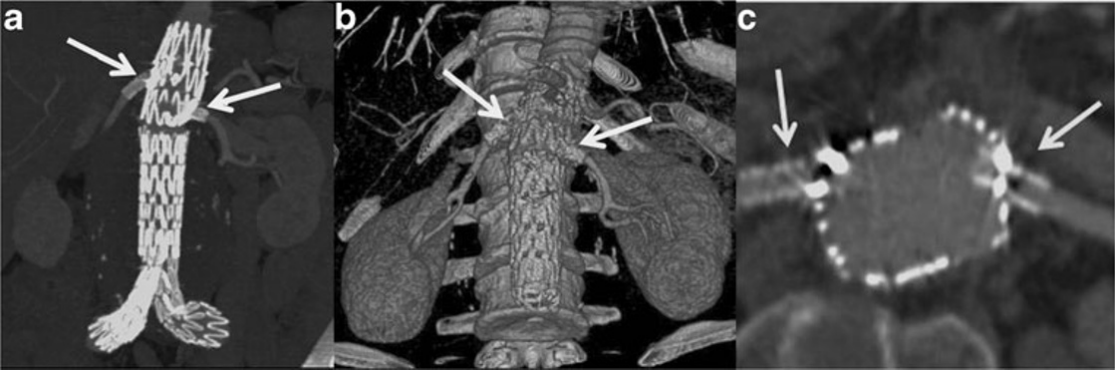
MIP (**a**) and VRT (**b**) images show a fenestrated stent-graft with a covered metallic portion that incorporates the renal arteries, which are made patent by the associated placement of covered stents (**c**)

An aortic stent-graft is a device composed of a metallic portion (Nitinol, Elgiloy, and stainless steel) and graft material (Polyester, PTFE). On CT images only the metallic portion is visualized. On the basis of the general morphology, there are three types of stent-grafts available for treating abdominal aortic aneurysms: straight, aorto-uni-iliac grafts and bifurcated. Straight aortic tube grafts have the proximal and distal attachment sites in the aorta, above the aortic bifurcation (aorto-aortic). The aorto-uni-iliac device is a stent-graft that is deployed from the supra-aneurysmal aorta to one iliac artery only; the opposite iliac artery is then occluded with an endovascular occlusion device in order to prevent retrograde blood flow into the aneurysm sac, and a femoro-femoral crossover graft maintains blood flow into the opposite limb (Fig. 2). Bifurcated stent-grafts are extended to the iliac arteries. On the basis of proximal fixation, stent-grafts are also classified into infrarenal/suprarenal fixation and fenestrated devices. The infrarenal stent-graft is placed just below the caudal renal artery. The suprarenal fixation device has an uncovered metallic portion placed above the ostia of the renal arteries and a covered portion placed below the renal arteries: the radiological markers placed between the uncovered and covered portion are represented by two metallic points located on the stent-graft metallic structure at the opposite position [Fig. 3]. The fenestrated device has a covered metallic portion of the graft that incorporates any visceral vessels such as the renal arteries, superior mesenteric artery or celiac trunk; the patency of the incorporated vessels is maintained by the associated placement of covered stents (Fig. 4).

## Patency

**Fig. 5:**
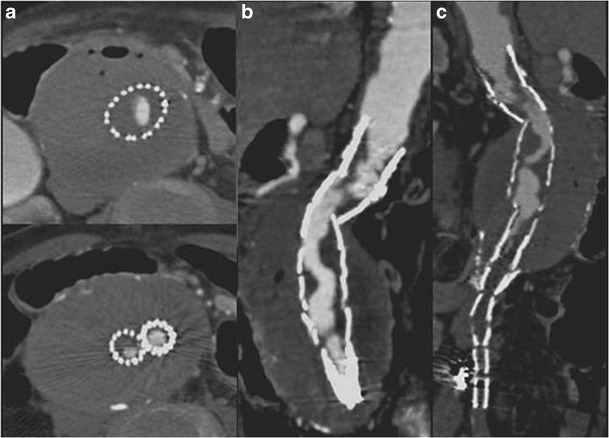
Axial (**a**) and CPR (**b**–**c**) images show a stent graft thrombosis causing significant intraluminal stenosis

Stent-graft and distal branches are considered patent if a uniform and homogeneous contrast enhancement is detected within them. On the other hand, graft thrombosis is recognized as an intraluminal, concentric or eccentric hypodense area within the stent-graft on CT images obtained after contrast-media injection. Angulation of the prosthesis could lead to a stenosis or thrombosis with consequent reduction of either the inflow or outflow, with increased possibility of in-stent thrombosis and stenosis, which can be manifest in about 3–19 % of cases [8, 9]. This thrombotic apposition could progressively lead to complete occlusion of the graft. Graft occlusion appears as a complete non-enhancing intraluminal area within the stent-graft. Graft limb occlusion may be directly due to compression of the limb in the proximal neck or in a narrow distal aneurysm neck, or to kinking or compression of the limb in a narrow or tortuous iliac artery. In case of iliac limb thrombosis, a femoro-femoral cross-over graft can be made, whereas a main body occlusion needs a complete surgical conversion. These complications are adequately visualized on both axial and 3D images (Fig. 5).

## Integrity

**Fig. 6:**
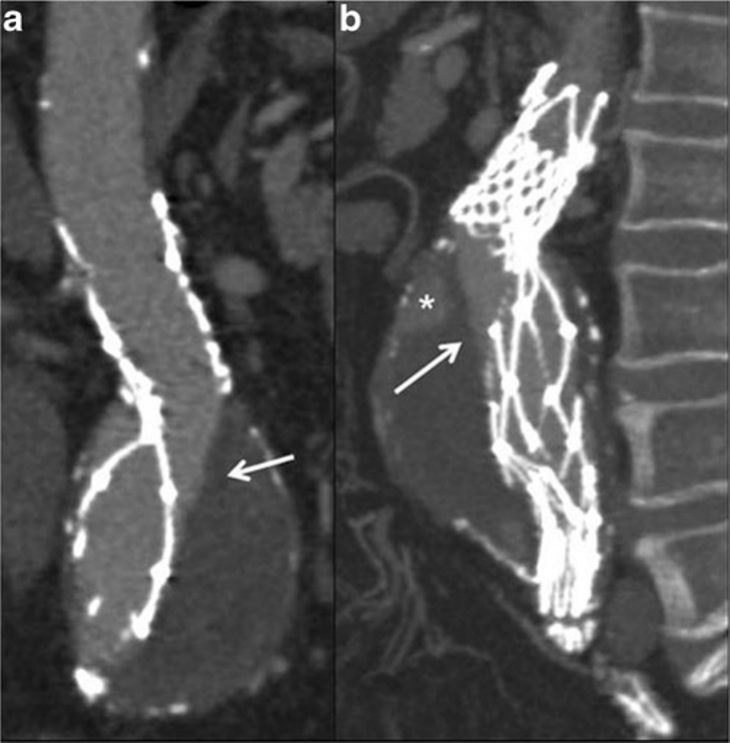
MPR (**a**) and MIP (**b**) images show a distortion of the metallic stent-graft structure. The integrity of the graft material as demonstrated by the linear and defined margin of the contrast-medium inside the stent-graft needs to be observed (arrows). A type-II endoleak is also detected (* in **b**)

Fractures or distortions of the metallic stent-graft structure are rare but important complications: these complications are better visualized on 3D rather than axial images (Fig. 6). Another complication that must be promptly recognized is stent-graft migration that occurs because of poor attachment of the stent to the aortic wall, which can cause sac reperfusion and subsequent aneurysm rupture.

## Position of the stent-graft

**Fig. 7:**
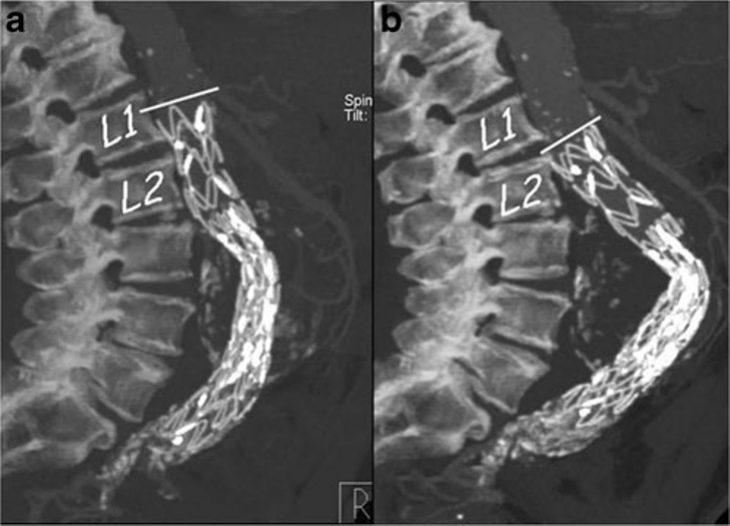
One month (**a**) and 1 year (**b**) post-EVAR follow-up sagittal MIP images. At 1 year follow-up the stent graft appeared to have migrated distally with a significant angulation of the main body

Stent-graft migration usually requires urgent treatment. Unless the graft can be extended proximally, treatment is usually carried out by conversion to open repair. Only migration of more than 4 mm is generally considered significant. This is related to a number of factors, including inadequate proximal fixation (incorrect sizing, conical-shaped neck, short neck and angulation of the neck), progressive dilatation of the proximal neck and aneurysm size, as well as iliacal fixation [10–12]. MDCT angiography clearly detects minimal stent-graft migration: the diagnosis is based on the comparison of 1-month follow-up CT images evaluating the relationship between the proximal end of the stent-graft and lumbar vertebral body or renal artery origin on 3D and axial images, respectively (Fig. 7).

## Sac aneurysm changes

Since aneurysm exclusion is expected to be correlated with shrinkage, surveillance of aneurysm dimensions is mandatory for asserting the adequacy of aneurysm exclusion from the blood flow and for guiding the need for reintervention in selected cases. The aneurysm volume may increase slightly immediately after EVAR (3–4 mm), given the additional volume and the external force exerted by the stent-graft or, alternatively, the swelling of the aneurysm sac induced by perigraft thrombosis [13]. On the other hand, the volume tends to increase if sac perfusion is present or decreases during the follow-up in the absence of endoleaks. Diameter measurements are most accurate if performed on axial images perpendicular to the aortic long axis, but volume assessment has been proven to be more accurate than diameter in the early detection of aneurysm growth; however, volume assessment is time-consuming and requires advanced processing, dedicated equipment and skilled operators [14].

## Endoleak

**Fig. 8:**
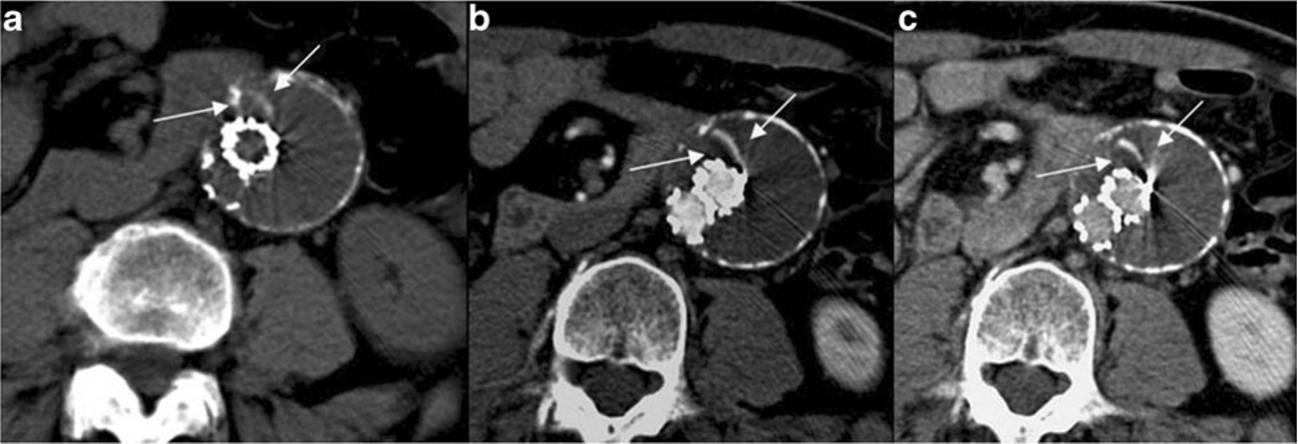
Axial 12-month follow-up MDCT images showing an AAA treated with EVAR. The combined analysis of unenhanced (**a**), arterial (**b**) and late-phase enhanced images (**c**) allows the correct characterization of the thin hyperdensity within the sac (arrows) as a linear calcification

Endoleak is defined as a persistent blood flow within the sac excluded from the stent-graft, and it occurs in 2.4–45.5 % of patients after EVAR [15]. On CTA images, the endoleak appears as a high attenuation area outside the graft but within the aneurysm sac, detected on arterial and/or delayed phase images, but generally absent on unenhanced images. Unenhanced images can be helpful to avoid false-positive diagnoses, allowing the differentiation of calcifications in the aneurysm sac from an endoleak (Fig. 8). Because endoleaks have variable flow rates, they can be detected at variable times after contrast material injection. For this reason, a delayed phase has been recommended: in detail, this phase could detect endoleaks not visualized during the arterial phase, the so-called low-flow endoleaks. Early endoleaks occur in the first 30 days following EVAR, while endoleaks that fail to seal within 30 days are called persistent endoleaks. Proper classification of an endoleak is important for its subsequent management [16, 17].

**Fig. 9:**
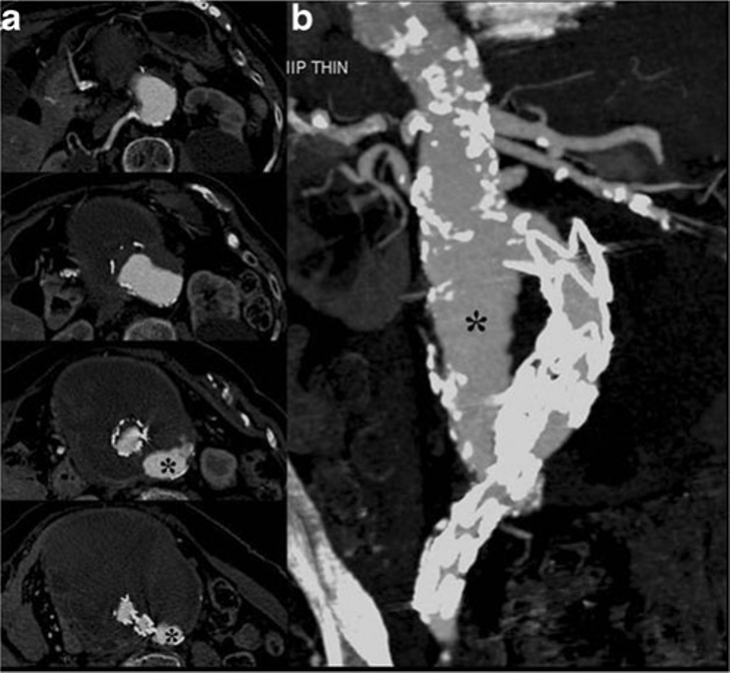
Axial (**a**) and MIP- (**b**) images show the stent-graft caudal migration with subsequent proximal type-I leak (*)

**Fig. 10:**
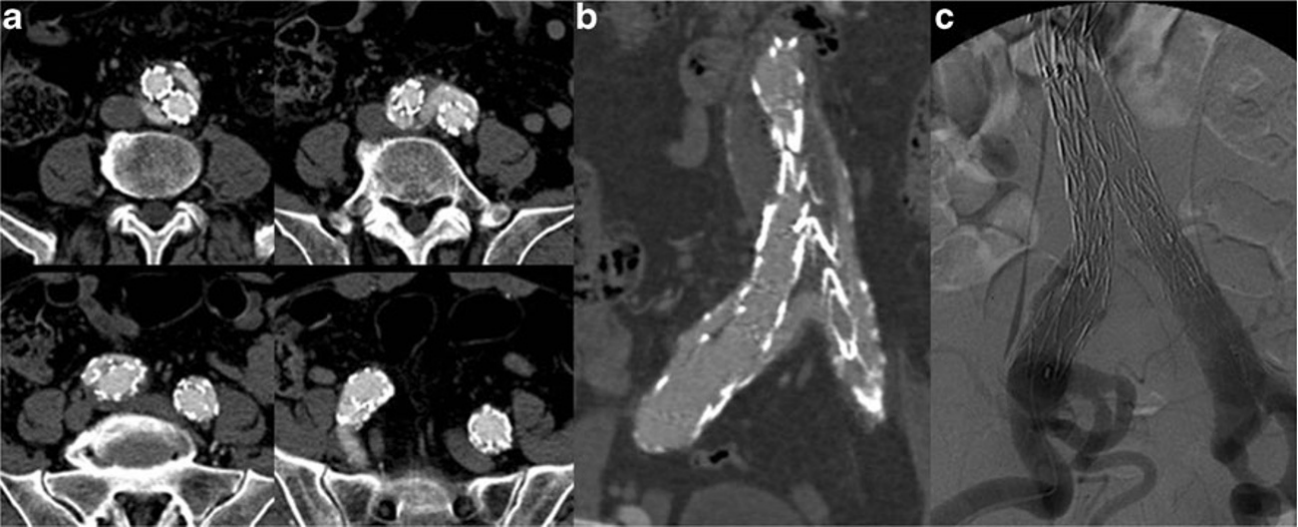
Axial (**a**) and CPR (**b**) images show an incomplete distal attachment of the right iliac limb causing a type I leak, as confirmed by DSA (**c**)

**Fig. 11:**
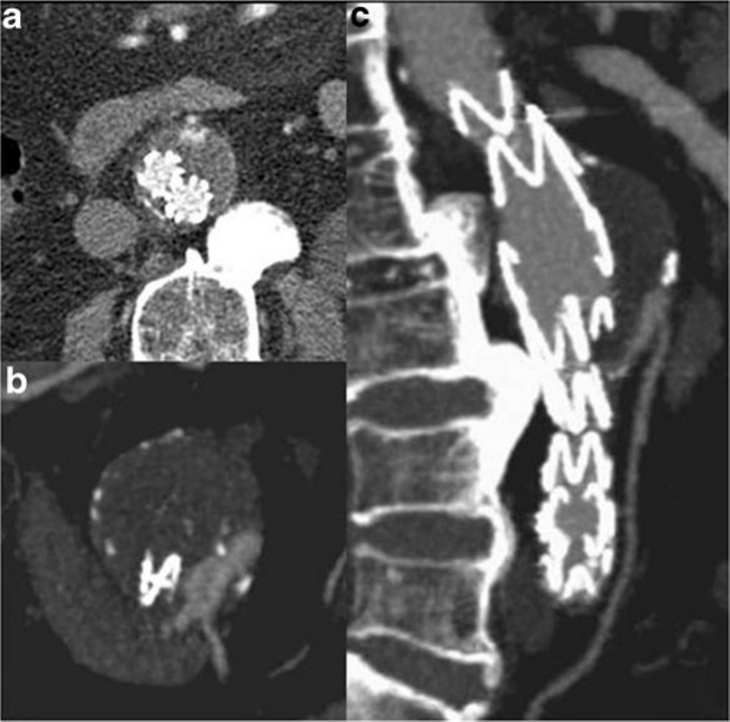
Type II endoleak. Axial (**a**) and thin MIP (**b**, **c**) images show back-filling of the aneurysm sac through the inferior mesenteric artery

**Fig. 12:**
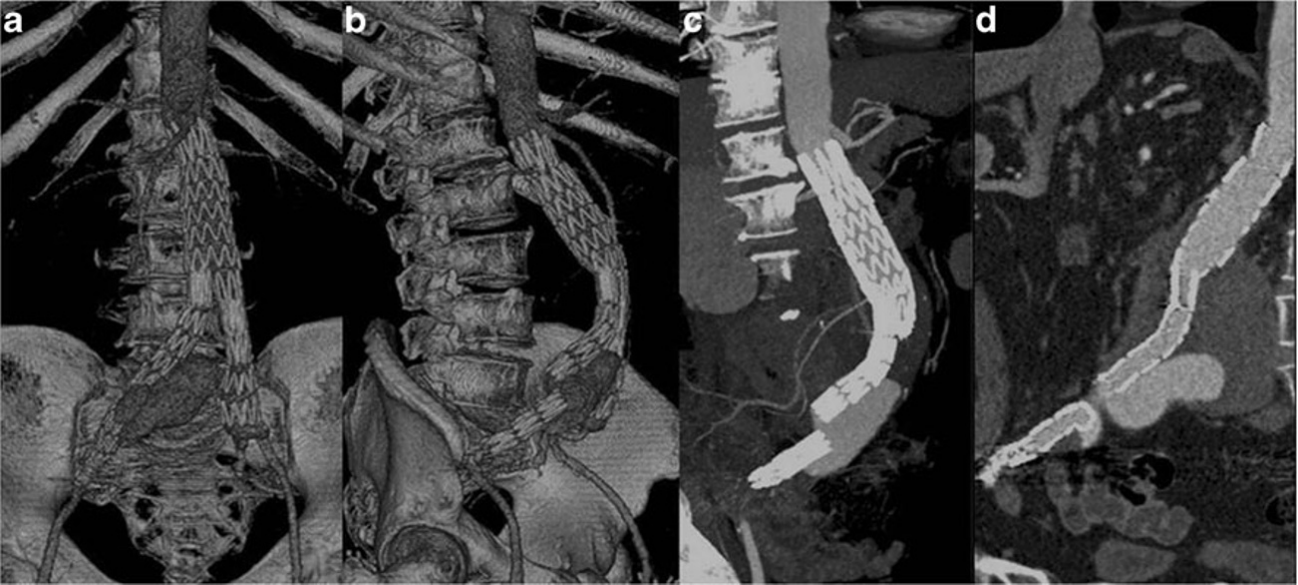
VRT (**a**, **b**), MIP (**c**) and CPR (**d**) images show a type III endoleak due to a right iliac limb disconnection and distal migration

**Fig. 13:**
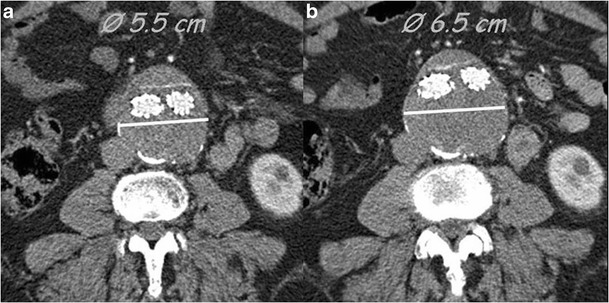
Type V endoleak; axial images. Follow-up MDCT examinations performed at 6 months (**a**) and 12 months (**b**) show an increase in sac diameter without evidence of endoleaks

- Type I is caused by separation of the device from the arterial wall, resulting in leaks originating at the proximal and/or distal attachment sites of the graft because of a technical (e.g., suboptimal stent-graft diameter) or anatomical (e.g., a short, irregular, ulcerated or angulated landing zone without an optimal conformation of the stent-graft to the curved aortic contour) problems, or to its caudal migration. On CTA images, it often appears as a huge and circumferential leak, adjacent to the proximal or distal end of the prosthesis (Figs. 9 and 10).
- Type II endoleaks are caused by back-filling of the aneurysm sac via branch vessels, such as the lumbar arteries and inferior mesenteric artery excluded by the stent-graft. On CTA images, the type II endoleak is most pronounced at the periphery of the aneurysmal sac, with little or no contact with the prosthesis, is commonly located in a posterior or lateral position, and is associated with opacification of the lumbar arteries. If an endoleak is located in the anterior position, a retrograde flow into the sac by the inferior mesenteric artery must be suspected (Fig. 11).
- Type III endoleaks arise from a fabric tear, modular or graft disconnection, and are more likely when multiple prostheses with short overlapping areas are used. On CTA images, the leak is strictly adjacent to the prosthesis, with little or no contact with margins of the aneurysmal sac, without opacification of the lumbar arteries or inferior mesenteric artery (Fig. 12).
- Type IV involves vascular flow caused by the high porosity of the graft, most likely created by the numerous suture holes holding the graft material to the stent. They are usually only detected on conventional angiograms performed at the end of the procedure.
- Type V or endotension refers to a growth of the aneurysm sac but without demonstrable reperfusion defects (Fig. 13).

Type I and type III endoleaks require prompt treatment as they are associated with a high risk of sac rupture. However, the most common endoleak found in endovascular stent-grafting is the type II endoleak.

## Renal artery patency

**Fig. 14:**
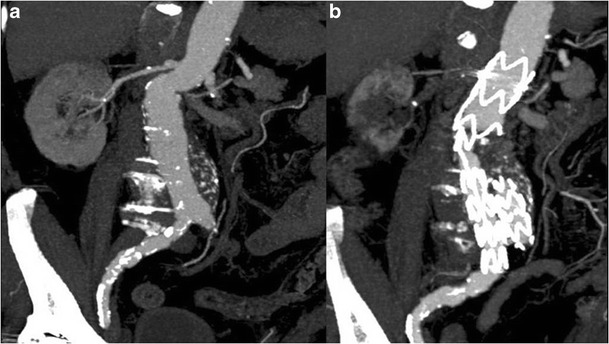
MIP images obtained before (**a**) and after (**b**) EVAR. The follow-up MDCT examination revealed a significant proximal right renal artery stenosis. The right kidney appeared decreased in size and showed an area of parenchymal hypoperfusion due to infarction

Renal artery stenosis, occlusion and dysfunction represent the main concern with EVAR, especially in patients with inadequate anatomy of the proximal aneurysm neck, pre-existing renal disease or in whom suprarenal fixation was used. Axial CT images are commonly used to monitor the patency of these arteries following stent-graft placement and to detect renal infarction following suprarenal stent grafting. However, both MIP and CPR are useful and complementary to axial images for the detection of stenosis and occlusions because of the tortuous course of the renal arteries. The additional views provided by CT angiography allow displaying the renal arteries in multiple planes and projections, which is often necessary for depiction of stenosis. In renal infarction, contrast-enhanced CT scans demonstrate a sharply demarcated, wedge-shaped area of decreased attenuation in the kidney [18] (Fig. 14). However, to date no significant correlation has been found between EVAR and renal impairment [19–20].

## Access site complications

**Fig. 15:**
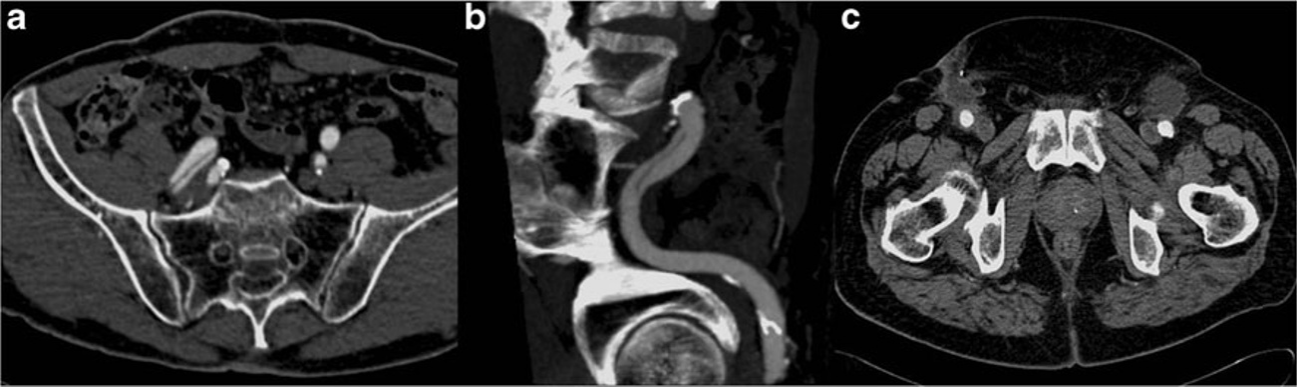
Axial (**a**) and MIP (**b**) images show an endoluminal hypodense line within the right external iliac artery representing a dissection; **c** axial image shows bilateral small hematomas at the femoral incision site

Usually a transfemoral approach is used to perform EVAR, exposing the femoral artery by a vertical groin incision. The large-caliber delivery sheaths used for EVAR, up to 26 Fr, increase the morbidity rate compared to the usual catheter size used for routine diagnostic angiography. The minimum vessel diameter required to allow passage of the EVAR device is 7–8 mm. Access site complications include dissection, demonstrated by a linear endoluminal hypodense area (intimal flap) (Fig. 15a–b) or arterial rupture, with contrast material extravasation outside the artery. Other complications are pseudoaneurysms, especially close to the entrance site, hematomas near the femoral incision, demonstrated by a high-attenuating area (Fig. 15c), infections and lymphoceles [21].

## Conclusion

MDCT is the method of choice both in the pre- and postoperative setting of EVAR procedures. A better understanding of the procedure followed, the devices used, the normal postoperative imaging features and the possible procedure-related complications ensures an optimal planning and follow-up of patients who undergo an EVAR procedure. Finally, in order to better determine the long-term performance of these devices, strict surveillance of all patients is mandatory.
